# Rafts, Nanoparticles and Neural Disease

**DOI:** 10.3390/nano2030217

**Published:** 2012-08-06

**Authors:** Vishal Gulati, Ron Wallace

**Affiliations:** 1 Ross University School of Medicine, Miami Beach Community Health Center, 11645 Biscayne Boulevard, North Miami, FL 33181, USA; Email: vgulati@knights.ucf.edu; 2 Department of Anthropology, University of Central Florida, Orlando, FL 32816, USA

**Keywords:** rafts, nanoparticles, nanomedicine, neural disease, epilepsy, Parkinson’s disease, Alzheimer’s disease, liposomes, solid lipid nanoparticles

## Abstract

This review examines the role of membrane rafts in neural disease as a rationale for drug targeting utilizing lipid-based nanoparticles. The article begins with an overview of methodological issues involving the existence, sizes, and lifetimes of rafts, and then examines raft function in the etiologies of three major neural diseases—epilepsy, Parkinson’s disease, and Alzheimer’s disease—selected as promising candidates for raft-based therapeutics. Raft-targeting drug delivery systems involving liposomes and solid lipid nanoparticles are then examined in detail.

## 1. Introduction

In June 1844, Claude Bernard demonstrated that the alkaloid curare, introduced beneath the skin of a frog, produced paralytic effects. His student Vulpian later proposed that the toxin blocked impulse transmission at the neuromuscular junction [1]. This landmark experiment signaled the emerging recognition that the action of a drug occurs at specific cellular locations [2]. Since Bernard’s day, pharmacology has been focused on the synthesis, optimization, and clinical application of cell-specific drug delivery systems. During the last decade, however, the platform has been extended to sub-cellular targeting strategies [3,4]. A major dimension of the new approach is drug delivery to membrane rafts: Mesoscopic molecular ensembles of sphingolipids and cholesterol with linear dimensions from 20 to 200 nm, and lifetimes ranging from 10^−2^ to 10^3^ s. The rationale is straightforward: Rafts, as ordered 2D structures dispersed in a fluid environment, help regulate intracellular traffic by facilitating interaction of associated signaling proteins, and by playing significant roles in endocytosis and protein sorting [5,6]. Accordingly, raft-targeted drugs which could manipulate these features could potentially be directed to a subcellular site of pathology [7,8]. In a separate but closely-related development, researchers in nanomedicine—the engineering and manipulation of atomic and molecular devices for therapeutic and imaging applications—are formulating nanoparticles for use as drug carriers [9]. The unifying goal of this effort is a biocompatible nanoparticle equipped with a cell-targeting ligand and, coupled or separately, a cell-penetrating peptide, which could release a potent drug at a targeted molecular site with high bioavailability [10]. A synthesis of these strategies appears increasingly likely: Raft-mediated drug delivery utilizing nanocarriers may soon emerge as a major component of molecular medicine.

This review article examines recent research, continuing controversies, and the possible clinical use of raft-targeting nanoparticles as a drug delivery system in the treatment of neural disease. (Gene therapies involving nanoparticles are beyond the scope of this review.) It begins with a fundamental issue: Do rafts exist *in vivo*, or are they methodological artifacts? To evaluate this question, the strengths and weaknesses of the major laboratory techniques utilized in raft research are discussed at length. Early strategies for raft observation, *i.e.*, detergent extraction and cholesterol depletion, are contrasted with more recent biophysical approaches such as fluorescence resonance energy transfer, fluorescence recovery after photobleaching, and single-particle tracking. It is proposed that the highly varied time and space scales addressed by these techniques, and correspondingly varied raft properties, suggest a heterogeneous, functionally differentiated membrane that mediates complex inputs at widely ranging orders of magnitude. The article then considers the role of rafts in neural disease, evaluating three disorders in depth, epilepsy, Parkinson’s disease (PD), and Alzheimer’s disease (AD), rather than a large number summarily. The three were selected in view of their relatively high morbidities, devastating physiological, psychological, and behavioral effects, raft etiological involvement, and probable suitability for nanoparticle-based raft targeting. In each of the three cases, evidence is presented for raft localization of malfunctioning proteins—potential drug targets—implicated in the disease. Nanoparticle drug carriers are then discussed at length. Emphasis is placed on two major classes of lipid-based nanoparticles (LBNs), liposomes and solid lipid nanoparticles (SLNs), based on several criteria: The increasing refinement of their fabrication techniques, their drug loading efficiencies, their ability to be coupled with site-specific targeting ligands, their drug release potential, and, most significantly, their minimum toxicity. The article then examines recent experimental studies of LBN drug delivery in epilepsy, PD, and AD. These discussions form the basis for suggested new approaches in LBN raft targeting. The hypothetical systems involve active LBN targeting of raft-associated receptors, and subsequent drug release and targeting of raft-associated, and hence more stabilized, proteins implicated in the disease. It is emphasized that the proposals confront serious obstacles, e.g., the identification of receptor-subunit conformations specific to each disorder. Nonetheless, it is concluded that the increasing appeal of interdisciplinary approaches and use of computational models will likely overcome these barriers and yield effective therapies.

## 2. Methodological Issues: Do Membrane Rafts Exist?

In this section, a subset of methods of raft investigation is outlined and evaluated. Following a brief overview of detergent-extraction and cholesterol-depletion techniques, there is a more extensive examination of biophysical methods. It should be noted that several approaches in addition to the ones discussed here are being increasingly deployed in raft studies. These include: Investigations of the caveolin protein, believed to be enriched in rafts, and which may play a significant role in synaptic development and neuronal signaling [11]; co-immunoprecipitation studies, recently utilized to examine possible raft-specific-protein co-distribution in photoreceptors [12]; electron microscopy, which has played a major role in investigating putative raft association, and possible raft modulation of function, of the glycine transporter [13]; and, perhaps most importantly, atomic force microscopy, which may ultimately provide direct evidence of rafts in native membranes [14]. A detailed examination of all methods used in raft studies would require a separate review. Therefore this section emphasizes approaches that, for the last 20 years, have been at the center of theoretical debate regarding raft *in vivo* existence. 

### 2.1. Detergent Extraction and Cholesterol Depletion

Following more than two decades of vigorous methodological debate, the *in vivo* existence of rafts and their role as modulators of protein signaling dynamics remain controversial [15,16,17]. The structures are operationally defined as detergent-resistant membranes (DRMs), based on an historical experiment conducted by D.A. Brown and J.K. Rose in which sphingolipids and glycosylphosphatidylinositol (GPI)-anchored proteins were found to be insoluble in cold detergent (Triton X-100), and floated to the top of a sucrose density gradient as a cholesterol-dependent fraction [18]. In the years since their investigation, extensive replications and the development of alternative detergent-extraction methods using CHAPS, NP-40, octylglucoside, as well as lowered concentrations of Triton X-100, have not removed the skepticism that greeted the original study [19,20]. The various extraction techniques produce membrane fractions differing somewhat in their protein and lipid content. This raises the possibility that rafts may be artifacts: Each raft a unique product of the technique by which it is made. The use of detergent-free protocols, such as the lysis of whole cells in a sodium carbonate buffer (pH 11), has also been reported, but presents a different problem. The final DRM fraction obtained through centrifuged sucrose gradient includes both plasma and intracellular membranes. Since the latter also contain rafts, the cellular source of a DRM-fraction component cannot be determined with certainty, thereby compromising the method’s usefulness. Cholesterol depletion, another widely used technique, is also problematic. Because cholesterol association with sphingolipids is essential, by definition, for raft formation, depletion via sequestration using cholesterol-binding compounds, removal from the membrane by methyl-β-cyclodextrin application, or through inhibition of cholesterol biosynthesis should disrupt cellular functions believed to be raft-dependent. The disruptive effects of cholesterol depletion, however, may not be confined to rafts. The method also disrupts the cytoskeleton, generating pleiotropic effects on morphology, exocytosis, and intracellular signaling. In the neuron, for example, filamentous actin (*F*-actin), a component of cytoskeleton “fences” concentrated at post-synaptic membranes, slows protein lateral diffusion, permitting stabilization of transmitter receptors. *F*-actin disruption through cholesterol depletion removes a significant obstacle to protein lateral mobility, permitting the proteins to move more rapidly into and out of synapses, an effect that would likely compromise effective synaptic transmission. In response to these critiques, proponents of the method contend that partial cholesterol depletion (~50%) from the *exofacial* leaflet will disrupt raft function within 10 min with no effect on other cellular functions. Yet even if this is the case, raft regulatory functions on the inner membrane leaflet, a raft property which would appear especially likely in intracellular signaling, are not addressed by the method. 

### 2.2. Biophysical Methods

Skepticism regarding DRM and cholesterol depletion has motivated many researchers to develop biophysical methods which can reveal molecular interactions consistent with membrane rafts while not disrupting other cellular structures [21,22]. Several techniques are presently in use, including fluorescence resonance energy transfer (FRET), fluorescence recovery after photobleaching (FRAP), and single-particle tracking (SPT). The FRET method is based on radiationless energy transfer from an excited donor fluorophore to an acceptor fluorophore, resulting in a reduction of the donor’s fluorescence intensity, and an increase in the acceptor’s emission intensity. Because energy transfer can only occur at intermolecular distances of 3–6 nm, FRET has been extensively used for investigating the possibility of probe co-localization in rafts. Applications of the method have yielded contrastive results. For example, negative results were obtained in an early (1998) FRET study of MDCK cells using as a probe system labeled antibodies against the GPI-anchored protein 5' nucleotidase (5' NT) [23]. Although the investigators did not rule out the possibility that some 5' NT was clustered in rafts, most of the probes were randomly distributed across the cell surface. By contrast, another FRET study conducted that same year, which measured the energy transfer between isoforms of the folate receptor bound to a fluorescent analogue of folic acid, detected membrane heterogeneities consistent with the raft model [24]. In an attempt to resolve the issue, raft investigations have increasingly utilized FRAP. In this method, all the molecules in a membrane region are labeled with fluorescent tags. The region is then irreversibly photobleached with a laser pulse, causing the fluorescence lifetime of the tagged molecules to quickly elapse. The optical effect is a uniformly fluorescent field containing a single dark spot. As the membrane is monitored, the still-fluorescing probes diffuse into the bleached area until a steady state is reached. The rate of fluorescence recovery, as determined by repeated images recorded at low laser power, is a measure of the fraction of membrane molecules capable of diffusing freely (the mobile fraction *M_f_*). By means of mathematical modeling, parameters such as *M_f_*, the diffusion coefficient *D*, and rates of fluorophore binding and unbinding with an insoluble scaffold such as a raft or the cytoskeleton, yield a dynamic interpretation of the photobleached region. The choice of an appropriate model, however, has been the object of much controversy, involving the relative significance of binding kinetics, diffusion, and the species of photobleached molecule. As a consequence, despite the development of new confocal FRAP techniques in which only one point in the sample is illuminated at a time, thereby improving image resolution, the best means of interpreting the data remains a topic of active debate.

Mathematical issues notwithstanding, FRAP investigations of rafts are being conducted extensively. The use of influenza hemagglutin (HA) mutants as indicators of protein association with rafts is gaining increasing acceptance as a preferred FRAP strategy. Studies of HA sorting to the apical surface of epithelial cells indicate that the HA transmembrane (TM) sequence determines its raft- or non-raft association [25]. Building on these studies, it was found that the latter property could be manipulated via point mutants of the TM sequence, ultimately permitting comparison of proteins that do (or do not) partition into rafts in the plasma membrane [26]. Importantly, separate studies have shown that Ras proteins—GTPases localized in the inner membrane leaflet which regulate cell proliferation, differentiation, and survival—are also characterized by differential raft targeting due, in this case, to variable lipid anchors. The combined FRAP study of HA proteins and Ras isoforms thus permits the investigation of coupling between inner and outer membrane leaflets, potentially significant in transbilayer signaling and approaches in molecular medicine [27]. A recent study based on this rationale monitored the effects of clustered and cross-linked HA proteins in the external leaflet on the lateral diffusion of green fluorescent protein (GFP)-tagged Ras proteins in the inner leaflet [28]. As anticipated, diffusion was most strongly affected by the raft-interacting HA proteins, while the non-raft mutants had no effect. Moreover, HA clustering modulated Ras cytosolic signaling: The association of inner-leaflet proteins (e.g., GDP-loaded wild type [wt] H-Ras, a precursor to GTP-loaded H-Ras[wt]) was enhanced through raft stabilization, while the next step in the signaling process (Erk phosphorylation ,which requires H-Ras-GTP dissociation) was inhibited. Based largely on these findings, the investigators suggest a generalized model in which extracellular ligands bind with outer-leaflet raft-localized receptors, inducing cross-linking, stabilization of inner-leaflet raft-associated assemblies, and modulation of downstream signaling. Therapeutic implications are, as noted, potentially considerable, but these and other FRAP studies would be significantly strengthened by a consensus mathematical model. 

Paralleling the theoretical insights obtained from FRAP, single-particle tracking (SPT) studies, in which the trajectory of a single molecule can be closely monitored and is not masked by population averaging, are yielding intriguing evidence of membrane organization. The method essentially involves labeling individual molecules with latex beads or colloidal gold particles and rapidly imaging the signal originating from incident light scattered by the bound particles [29]. The consecutive images are then connected to define particle trajectories. The latter determination involves computer-assisted statistical algorithms such as Monte Carlo methods; these are useful in modeling phenomena such as molecular motion in which there is a large number of coupled degrees of freedom. The SPT approach, although very powerful, is not without its drawbacks. Apart from the inherent trade-off between spatial and temporal resolution, the statistical analysis is most informative when a high number of particles is tracked in each experiment. Moreover, the latex bead, larger by several orders of magnitude than the molecule to which it is bound, may interact with obstacles in the extracellular matrix, significantly slowing the molecule’s motion and biasing interpretation. As well, due to noncovalent bonding, it is possible for a bead to escape and bind to another molecule. These limitations have motivated the increasing use of organic dyes or fluorescent proteins which are less invasive than latex or gold. 

SPT studies of the plasma membrane have altered FM to a clearly dramatic extent, perhaps justifying the recent claim of the Kusumi Membrane Organizer Project that the changes amount to a “paradigm shift” [30]. Kusumi’s group proposes that the plasma membrane is comprised of submicron compartments (“corrals”), varying is size from 30–230 nm, defined by actin cytoskeleton fences anchored by transmembrane protein “pickets”. High-speed tracking (40,000 frames/s) of L-α-dioleoylphosphatidylethanolamine (DOPE) revealed distinctive non-Brownian (suppressed) diffusion trajectories. Computer software designed in the Kusumi lab extrapolated the possible compartment sizes and detected those instances in which the diffusion coefficient was “suddenly and briefly increased”. These instances were interpreted as movements of the labeled particles over a cytoskeleton picket fence separating adjacent compartments, or “hop diffusion”. In an alternative version of the model proposed by A. Tsuji and S. Ohnishi, monomers move between compartments through cytoskeleton “gates” created by the dissociation of the spectrin tetramer into two dimers (open state) and subsequent re-association (closed state) [31]. Possibly the most significant aspect of the Kusumi model, particularly for drug delivery, is “oligomerization-induced trapping”, in which molecular complexes move more slowly than monomers across the intercompartmental boundary, and are more likely to be tethered to the cytoskeleton. As a result, the complexes are temporarily localized within a membrane compartment, perhaps enhancing local coordination of extracellular and cytoplasmic signals. 

### 2.3. Raft Sizes and Lifetimes: Toward a Rapprochement

As the above overview indicates, despite two decades of research examining the membrane liquid-ordered state, there is still significant disagreement regarding the dimensions, lifetimes, functions, and even the existence of membrane rafts as well as the methods applied to their study. There remain unresolved problems associated with experimental preparations (e.g., extent of cellular damage due to cholesterol depletion; instability of latex beads in SPT), and quantitative analysis (e.g., the best form of the Monte Carlo algorithm for interpreting raft dynamics). Beneath the controversies surrounding protocols and quantitative methods, however, there may be a deeper issue. Conspicuously lacking in much of the literature are synthesizing models extending over a wide range of temporal and spatial scales proposing how small, unstable domains (1.5–5 nm) identified in numerous studies may be functionally related to equally well-documented large, stabilized rafts (20–200 nm). Instead, “snapshots” of membrane dynamics, each characterized by a limited time and space scale, have become the inductive bases for competing raft models. 

**Figure 1:**
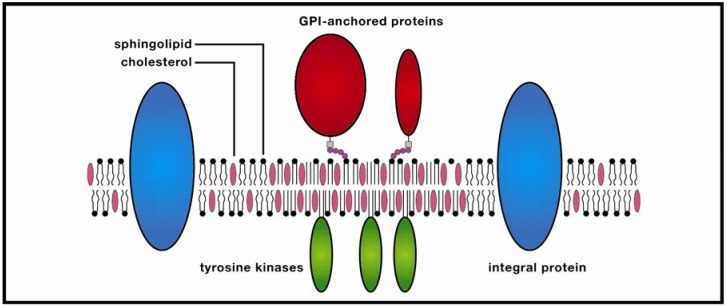
Membrane raft. These ordered molecular platforms comprised of sphingolipids and cholesterol play significant roles in endocytosis and signal-protein co-localization, both of which are potentially significant for drug-delivery technologies.

In addressing this explanatory impasse, it is useful to remember that ~3 billion years of cellular evolution have selected for a heterogeneous, compartmentalized, and functionally differentiated plasma membrane that mediates between external and internal inputs at a broad spectrum of time and space scales (Figure 1). The evolutionary picture is consistent with a highly optimized, poised system in dynamic equilibrium capable of rapidly shifting from nanodomains to rafts (*i.e.*, from ~10 to ~200 nm) in response to fluctuating local conditions [32]. The molecular basis of the system is the nonconformability of the cholesterol sterol structure with the rigid double bond of unsaturated lipids and the projecting amino-acid side chains of transmembrane proteins. These structural nonconformabilities induce the energetically more favorable exclusion of cholesterol from proteins and unsaturated lipids, resulting in cholesterol-enriched and protein-enriched domains. In the cholesterol-enriched region, glycosphingolipids and sphingomyelin are rarely present in sufficient concentrations to create large, stable rafts; as a consequence, the region is dominated by nanodomains. Could these nanodomains be functional? An intriguing hypothesis, proposed by Anderson and Jacobson [33], is that thermodynamically stable, cholesterol-enriched “lipid shells” ~7 nm in diameter and comprised of ~80 lipid molecules encase a transmembrane protein and reduce its buoyant density, thereby increasing its affinity for sphingolipid-cholesterol rafts. In defense of their model, they cite an extensive body of literature documenting the lipid-binding properties of several integral and peripheral membrane proteins (e.g., caveolin-1, synaptophysin, NAP22, MARCKS, prion). In an important variation of this viewpoint, proposed by Fantini and Barrantes [34], lipid shells do not merely surround a protein; they influence its conformation. This possibility was initially suggested by a search for structural motifs involved in the interaction of human immunodeficiency virus-1 (HIV-1) and amyloid proteins with sphingolipids, and was later extended to other proteins via structural similarity searches supported by crystallographic data. These studies indicated a possible sphingolipid binding domain (SBD) for protein ligands, of which the critical functional feature is the π-electron cloud of an aromatic ring. In an unusual form of H bonding, the aromatic ring electrostatically interacts with the partial positive charge of a sphingolipid galactose ring, inducing interfacial alignment. Subsequent sphingolipid-protein interactions (possibly also involving cholesterol) alter the protein’s conformation, preparing it to bind with a peripheral or integral protein.

In the protein-enriched domain, large rafts (~200 nm) are formed due to cross-linking of concentrated transmembrane proteins (supported by studies of Thy1, a GPI-anchored protein, and the ganglioside GM3), which may be further stabilized by temporary anchoring to the cytoskeleton. The stabilized protein clusters (3–5 nm in diameter) exclude sphingolipids which in turn attract cholesterol from the cholesterol-enriched region, thereby forming small clusters, the precursors of larger rafts. In the Kusumi group’s model, these condensed complexes, in contrast to monomers, display reduced lateral diffusion due to oligomerization-induced trapping in a cytoskeleton compartment. The combined effect is a transiently-stabilized platform which promotes coordinated signaling between extracellular and cytosolic compartments, as well as co-localization of membrane signaling components. This dynamic model would appear to resolve much of a lengthy debate in which highly varying temporal and spatial orders of magnitude for rafts have been referenced. Yet as anyone familiar with the history of membrane studies is aware, any raft model should be viewed with healthy skepticism, and the recognition that future research could alter the model significantly.

## 3. Rafts and Neural Disease

Although the methodological debate will likely continue for some time to come, a growing body of researchers, persuaded that rafts exist or are at least plausible, are examining their possible roles in health, disease, and therapeutics [35,36,37,38]. The shift in emphasis is motivated by the striking range of diseases, extending from HIV-1 to prostate cancer to vascular disease, in which rafts may be implicated. In neurons, putative raft involvement in several major disorders appears to pathologically reflect basic signal-platform functions of mediating between external and cytosolic traffic, and modulating the process of electrochemical signaling. A full discussion of the raft mechanisms which may modulate neuron function is beyond the scope of this article. (For a recent overview see [39]). Here we simply note that rafts may play significant regulatory roles in synaptic transmission, action potential (AP) propagation, and membrane signaling to the nucleus. Unsurprisingly, pathological variants of at least one of these basic functions, and sometimes more than one, appear in the etiologies of the three disorders we discuss below: Epilepsy, Parkinsonism, and Alzheimer’s disease. These diseases were chosen from several candidates because of their relatively high morbidities, devastating physiological and behavioral effects, possible raft involvement, and potential suitability for raft targeting via liposomes and solid lipid nanoparticles. The molecular physiology of each of these diseases will be examined, and therapeutic approaches involving raft targeting via lipid-based nanoparticles (LBNs) will be discussed at length.

### 3.1. Epilepsy

The epilepsies are a family of brain disorders defined by sudden, disordered, and synchronous activity of neuron populations [40,41,42]. The abnormal neural activity is transiently manifested as impairment or loss of consciousness, psychic or sensory disturbances, and alternating contraction and relaxation of muscle groups. Patients with epilepsy frequently experience depression and recurring pain, social stigmatization, lower rates of academic achievement, employment, and marriage. A recent meta-analysis evaluating pooled data of the global incidence of epilepsy determined that the median incidence was 45.0/100,000 for high-income countries and 81.7/100,000 for low- and middle-income countries [43]. Until recently epilepsy’s prognosis was generally pessimistic, despite the growing availability of pharmacotherapies. For example, Rodin’s 1968 survey concluded that only 30% of patients ever achieved a 2-year remission [44]. Despite the study’s limitations (it was small-scale and hospital-based) its conclusions were widely echoed throughout the biomedical research community. During the last decade, however, the outlook has become more favorable, reflecting a dramatically increased understanding of the molecular and cellular mechanisms responsible for the disorder. Prominent among several molecular systems, including in particular ion channel isoforms, which may be implicated in epilepsy is the ionotropic glutamate receptor *N*-methyl-D-aspartate (NMDAR). Importantly, NMDAR may be raft-associated, suggesting the possibility of raft-based molecular therapies.

The NMDA receptor has been a focus of neuroscience research over the last 20 years because of its significance in memory formation and neural disease [45,46,47,48,49]. As a consequence, its physiology is relatively well-understood. Unique among neuron receptors, NMDAR is voltage- and ligand-gated, a “coincidence detection” feature essential to its role in memory. The channel is typically comprised of four subunit proteins (two NR1 subunits and two NR2 subunits) which are targets for the neurotransmitters glycine and glutamate. Glycine binds with the NR1 subunits, and glutamate binds with the NR2 subunits. Binding by glycine and glutamate, however, is not sufficient to open the NMDAR channel, which is occluded by Mg^2+^. For channel opening to occur, there must also be postsynaptic depolarization due to inputs from adjacent synapses (coincidence detection). Under these conditions, the Mg^2+^ block is relieved and ion influx (primarily Ca^2+^ and Na^+^) occurs. Importantly, NMDAR permeability to Ca^2+^ is approximately 10-fold that of Na^+^, consistent with the receptor’s role (in combination with other membrane Ca^2+^ channels and possible input from ER Ca^2+^ stores) in increasing intracellular Ca^2+^ as a basis for learning and memory. Elevated cytosolic Ca^2+^ generates a set of interacting cascades which target DNA transcription factors. A well-described example is cyclic AMP response-element-binding protein (CREB), activated in response to a wide variety of stimuli [50]. Following activation, CREB binds with CBP (CREB-binding protein), which initiates gene transcription. The products of CREB-mediated gene expression target active synapses, modifying synaptic morphology and electrophysiological activity. NMDAR-based neuron modification for learning also involves Ca^2+^ binding with calmodulin. Ca^2+^/calmodulin activates Ca^2+^/calmodulin-dependent kinase II (CaMKII) which modifies synaptic signaling through NMDAR phosphorylation—thus changing the channel conductance state—or through facilitating the insertion of new receptors into the membrane. 

NMDAR physiology underlying synaptic plasticity may also, if uncontrolled, generate hyperexcitability, excitotoxicity, and neural degeneration associated with epilepsy. Indeed it has been proposed that epilepsy may be situated on a “plasticity-pathology” continuum in which NMDAR activity associated with learning and memory may be dysfunctionally up-regulated, thereby triggering the disease [51,52,53,54,55]. Consistent with this viewpoint, surface expression of NMDARs is dramatically increased in epilepsy, thus increasing the extracellular glutamate concentration. Remarkably, glutamate, the major CNS excitatory neurotransmitter, is also a neurotoxin; therefore its rapid accumulation in the extracellular space produces the neural damage associated with epilepsy as well as a wide variety of neurodegenerative and psychiatric diseases. Despite the intuitive appeal of NMDA channel blockers as therapeutic measures, these efforts have met with limited success because channel block interferes with NMDAR’s functional role [56]. A more promising approach is suggested by a recent, pioneering study by Ying Zhang’s team involving the downstream regulatory-element antagonist modulator (DREAM) protein [57]. DREAM is a member of the neuronal calcium sensor (NCS) protein superfamily, all members of which regulate cell Ca^2+^ homeostasis [58]. In its Ca^2+^-free state, DREAM binds with specific DRE sites to repress the expression of several genes including the Na^+^/Ca^2+^exchanger protein NCX3, which reduces Ca^2+^ rise following neural excitation. Ca^2+^ binding with two of four EF hand motifs induces DREAM dimerization, abolishing its ability to bind to the DRE site, thus enhancing surface expression of NCX3. DREAM may also reduce cell Ca^2+^ concentration by acting outside the cell nucleus: The Zhang study determined that DREAM binds directly to the NR1 subunit of NMDAR in cultured hippocampal neurons. Importantly, DREAM-NR1 binding had a neuroprotective effect against excitotoxic injury. The investigators speculate that DREAM binding achieves this effect by reducing the surface expression of NMDARs, either through “promoting their endocytosis or preventing their surface delivery”. Most significant from the standpoint of anti-epileptic drug (AED) therapies was the demonstration that DREAM’s neuroprotective effects could be achieved through the cell-permeable peptide TAT-21-40, constructed according to the binding site of DREAM to NR1. Implicit in the discovery is the possible LBN targeted delivery of TAT-21-40 to the NMDAR-associated raft for subsequent endocytosis, drug release, and drug binding with the NR1 subunit. 

### 3.2. Parkinson’s Disease

Originally described by James Parkinson (1755–1824) in an 1817 article entitled “An Essay on the Shaking Palsy” [59], Parkinson’s Disease (PD) is a neurodegenerative movement disorder affecting 1%–2% of the population over 50, and 1.5 million people in the US alone [60]. The clinical features of PD recognized by modern physicians are remarkably similar to those identified in Parkinson’s essay: Resting tremor, muscle rigidity, bradykinesia (slowing of movement), and postural instability. Non-motoric symptoms include depression, hallucinations, apathy, and fatigue [61]. PD’s defining neuropathological feature is the profound and selective degeneration of dopaminergic neurons in the substantia nigra pars compacta (SNc) of the basal ganglia. Many definitions additionally include the presence of Lewy Bodies (LB), which are intra-neuronal deposits of lipids and proteins.

The role of genetic predisposition continues to be actively debated [62,63,64]. Only about 10 percent of PD patients have a family history of the disease (familial PD), a statistic which traditionally suggested that the remaining 90% (sporadic PD) may have a predominantly environmental etiology. Proponents of the latter view have identified the insecticide Rotenone, the herbicide Paraquat, and the fungicide Maneb as critical to PD etiology, based on extensive *in vivo* animal models [65]. But this view is being modulated by use of the genome-wide association study (GWAS)—an epidemiological method used to identify genetic variations which occur more frequently in patients than in controls—which suggests that the genetic component may be stronger than previously suspected. For example, two GWAS studies, one of an Asian population [66], and one of people from European ancestry [67], identified variants at the SNCA (a-synuclein) and LRKK2 (leucine-rich repeat kinase 2) loci as possibly implicated in the disease. In combination with other studies, the overall state of the art suggests that PD’s precise etiology remains to be determined, but is likely multifactorial: A mix of environmental triggers and genetic predispositions.

Many current PD therapies afford relief from clinical symptoms, but do not provide neuroprotection [68]. These include: Deep-brain simulation in which electrical impulses are delivered to the subthalamic nucleus and globus pallidus via surgically-implanted electrodes; and dopamine replacement therapy via administration of levodopa (L-Dopa). This limited efficacy is motivating a search for molecular therapies that can cure or even prevent the disease by targeting its upstream pathophysiology. One major focus of this strategy is the small protein α-synuclein (α-syn), abundant in Lewy Bodies, possibly raft-associated, and a significant factor in PD etiology [69,70,71,72,73,74,75]. In physiologic SNc neurons, α-syn functions as chaperone, helping regulate exocytosis and endocytic recycling. Dopaminergic neurons form ~500,000 synapses, often relatively far from the soma, and thus requiring local chaperones as a feature of their highly autonomous, quality-control machinery [76]. As in other types of neurons, transmitter release is executed by SNARE (soluble NSF attachment protein receptor) proteins: SNAP-25 and syntaxin comprising the t-SNARE complex located at the presynaptic plasma membrane, and synaptobrevin-2 (also known as vesicle-associated membrane protein 2, or VAMP2), localized in vesicles. Recent studies indicate a role for α-syn in orchestrating SNARE complex assembly and neurotransmitter release. This chaperone function is not yet well defined, but the initial stage of the process appears to be lipid-regulated: Artificial-membrane experiments suggest that α-syn, unfolded in its native state, may interact with vesicle rafts enriched in phosphatidylserine (PS) and polyunsaturated fatty-acid (PUFA) lipids, a “combinatorial code” which generates two α-helices at the α-syn *N*-terminus [77]. This conformation closely associates with vesicle-membrane curvature, facilitating α-syn binding at its carboxy terminus to the VAMP2 amino terminus, an essential early step in SNARE complex assembly.

Why does α-syn malfunction? The yeast model (*Saccharomyces cerevisiae*) has been highly valuable in evaluating the question because its fundamental cellular processes including (importantly) transcription, trafficking, secretion, and protein folding are highly conserved in mammalian cells. Moreover, the yeast genome is well characterized and amenable to manipulation. Based on this rationale, a landmark yeast analysis by Tiago Fleming Outeiro and Susan Lindquist outlined an etiology in which synchronous and progressively elevated expression of human wild-type (WT) α-syn and the A53T mutant induces dose-dependent cellular dysfunction resembling familial PD [78]. At low levels of expression, WT and A53T are delivered to the plasma membrane via the secretory pathway. However, at higher expression levels, WT and A53T bind with “stalled” vesicles which, at even higher dosage levels, form cytoplasmic lipid droplets. Some insight into possible mechanisms underlying stalled vesicles has recently been provided by solution NMR spectroscopy of α-syn lipid-binding kinetics. Christina Bodner’s team examined the resonance signatures of artificial unilamellar vesicles (approximating the size and curvature of those present in synaptic terminals) added to α-syn solution including PD mutations A30P, E46K, and A53T [79]. The underlying rationale was that NMR signal attenuation corresponded to the fractional population of α-syn engaged in phospholipid binding at a specific *N*-terminal residue location. They observed two binding modes, SL1 and SL2, the former defined by a short lipid-bound α-helix followed by dynamic disorder in residues ~25-140, the latter defined by lipid binding of the full *N*-terminal domain in the α-helical conformation, with 40 *C*-terminal residues flexible in solution. Most significant for PD etiology was their finding that the α-syn molecules in the SL1 binding mode, with its long stretch of disordered *N*-terminal residues, are vulnerable to self-association. Importantly, the susceptibility is increased by mutations A30P and A53T. In addition, Bodner’s group speculates that duplication or triplication of the SNCA gene would produce a similar effect via a “lipid-limited” condition which would favor the SL1 binding mode. Possible toxic consequences of SL1 binding kinetics include impaired exocytic machinery (*i.e.*, stalled vesicles), accelerated α-syn fibrillation, pore formation, vesicle-membrane permeabilization resulting in dopamine leakage, and mitochondrial-membrane binding leading to mitochondrial dysfunction and cell death in SNpc neurons. Importantly, the suggestion that mitochondrial dysfunction is a downstream consequence of α-syn fibrillation appears consistent with separate evidence for α-syn accumulation in mitochondrion complex I of PD patients [80]. For a different model see [81,82]. 

Alpha synuclein association with plasma-membrane rafts, clearly relevant to molecular therapies, has been insufficiently investigated. Several studies have shown that α-syn binds with artificial membranes, but *in vivo* binding is supported by relatively limited evidence. In a pioneering study, Doris Fortin’s group monitored the effects of raft disruption on GFP-labeled α-syn in cultures of rat hippocampal neurons [83]. Cholesterol and sphingolipid synthesis was blocked by mevalonic acid, mevastatin, and fumonisin B_1_, a possibly less destructive protocol than cell treatment with cyclodextrin (recall earlier cautionary discussion of cholesterol-depletion methods). Following treatment, GFP-α-syn was substantially reduced in synapses, suggesting raft-associated synaptic localization. While further studies along these lines are obviously essential, these findings are consistent with extensive investigations suggesting raft enrichment of the presynaptic-terminal membrane. Thus, the use of raft-targeting LBNs which could inhibit or even reverse α-syn fibrillation via endocytosis and cytosolic release of an α-syn-targeting molecule appears a possible therapeutic strategy. 

Several candidates for this approach are emerging, primarily as a result of *in silico* screening techniques utilizing genetic algorithms (GA). The GA method mimics the optimizing effect (“blind watchmaker” [84]) of natural evolution in which iterated genetic variation, due to mutation and chromosomal cross-over, in a population of organisms is subjected over time to the filtering effect of environmental selection. GA has been applied to “populations” (libraries) of proteins which bind to α-syn and inhibit fibrillation, with the objective of discovering the most functionally optimized variants. In the GA version developed by Koichi Abe’s group, a library of 150 peptide ligands which bind with a hydrophobic α-syn region vulnerable to fibrillation were first filtered by calculating their docking energies with the target region, and “survivors” were then subjected to cross-over of peptide sequences and introduction of point mutations [85]. Six rounds of this procedure identified 10 superior sequences which bind with the α-syn region and modulate fibrillation. Abe *et al.* propose that the method be applied to peptides synthesized by Omar El-Agnaf’s group corresponding to α-syn residues 64–100 [86]; the peptides interacted with full-length α-syn and inhibited its aggregation. The investigators caution that, while the findings may have significant therapeutic implications, delivery problems such as transport through the blood-brain barrier (BBB), generation of an immune response, and peptide sensitivity to proteolytic degradation will need to be overcome. 

### 3.3. Alzheimer’s Disease

In 1911, Alois Alzheimer published a case report entitled “On peculiar cases of disease at higher age” [87]. His patient, 51-year-old Auguste D., was the first reported case of Alzheimer’s Disease (AD). Today, AD is the most common neurodegenerative disease (PD is the 2nd most common), affecting ~13% of people over age 65 [88]. Although data from normal seniors shows AD is not an inevitable consequence of aging, advanced age is nonetheless the major associated factor; consequently, morbidity is expected to increase in tandem with an aging population. Anterograde amnesia (inability to form new memories) and impaired performance of daily tasks are the dominant symptoms of early AD. A highly progressive and variable course of neurodegeneration follows until the terminal stage, in which the ability to communicate may be entirely lost. Other common symptoms include social withdrawal and personality/mood changes. Not surprisingly, these symptoms strongly characterized Auguste D. Following post-mortem examination of Auguste D.’s brain, Alzheimer further documented his findings, describing “cerebral atrophy and changes in neurofibrils”. 

Since this landmark analysis was published, over 100 years of research, aided greatly by the development of high-resolution microscopy and imaging techniques, e.g., electron microscopy (EM), have enabled researchers to arrive at a general consensus on AD pathophysiology. One of AD’s molecular hallmarks is the presence of amyloid plaques, *i.e.*, deposits of insoluble amyloid-β (Aβ peptide, a product of amyloid precursor protein (APP), in the brain parenchyma [89]. Animal models partially lacking the APP gene demonstrate reactive gliosis and impaired locomotor activity, suggesting a role in normal neurophysiology [90]. However, the exact role of APP in humans is controversial. In one putative pathway, APP processing is mediated by two sequential proteolytic events, regulated by the β-secretase enzyme BACE1 (β-site APP cleaving enzyme) and a γ-secretase (presenelin complex). β-secretase activity yields a 99-amino-acid C-terminal fragment that is subsequently cleaved by γ-secretase to release Aβ fragments 36–43 amino acids in length [91]. The insoluble 42-amino-acid aggregates (hereafter designated Aβ are the principal pathological variant. In the other, more predominant pathway, APP is first processed by the α-secretase ADAM10 at amino acid 17 within the Aβ sequence, thus preventing Aβ production after γ-secretase cleavage [92]. Interestingly, ADAM10-mediated APP cleavage does not appear to be either neuron- or raft-associated. A delicate balance exists between the two APP-processing mechanisms such that α-secretase up-regulation leads to decreased Aβ production, and vice-versa [93]. Not surprisingly, BACE1-mediated APP processing results in increased neuritic plaques. In addition, it has recently been shown that there is an approximate two-fold up regulation of the BACE1 enzyme in AD brains [94]. The imbalance that favors Aβ production—the key feature of the “amyloid hypothesis”—suggests that Aβ peptide oligomers are the primary physiological culprit responsible for AD neurodegeneration, altered neural-membrane permeability, and chemo-attraction for cells that together generate a cascade of inflammation [95]. Aβ may also cause synaptic failure, consistent with the finding that levels of (plaque-mediated) synaptic loss appear proportional to increasing AD severity [96]. The crystal structure of BACE1 has been documented [97]; and its essentiality for amyloid formation has inspired the development of drugs that target the enzyme’s active site.

Much like PD, current AD therapies aim at alleviating symptoms rather than halting disease progression. Currently, approved drugs include only select acetylcholinesterase inhibitors and the NMDA receptor antagonist memantine. Recent candidates for drug therapies include secretase targeting and even vaccination, but such interventions have so far failed to demonstrate efficacy in clinical trials due to multiple near-lethal side effects [98]. Among the problems encountered were toxicity of individual drugs, transport through the BBB, *P*-glycoprotein-mediated drug efflux, and the difficulty of accessing the secretase active site. Ongoing clinical trials of new agents that may overcome these obstacles, as well as different therapeutic strategies such as aggregation blockers and neurotropin replacement, are currently underway [99]. In addition, encouraging results have recently been obtained via exploitation of lipid rafts. This strategy is based on evidence suggesting that amyloidogenic processing of APP is raft-associated. The model was first proposed by Robert Ehehalt *et al.* who showed that cholesterol depletion in N2a cell lines reduced β-secretase-mediated processing of APP into Aβ peptide [100]. Their work was based on Simons’ 1998 experiment demonstrating a possible link between cholesterol and APP processing [101]. Following the active pursuit of this hypothesis, it was found that all Aβ generating components were localized in cholesterol-rich lipid rafts. Indirect evidence came from studies of cholesterol modulation and inherited forms of AD, particularly the APOε4 allele, whereby high plasma cholesterol levels positively correlate with increased likelihood of developing AD. On intuitive grounds, reducing cholesterol would likely inhibit Aβ processing; statins would thus be an obvious therapeutic choice, a possibility supported by numerous studies. However, the benefit of statins in AD treatment remains controversial; clearly, additional studies are needed that demonstrate a stronger correlation between statins and cognitive improvement. 

Although the mechanism of APP and secretase co-localization in lipid rafts is understood only in outline, many different experiments, e.g., cholesterol/endocytosis modulation, antibody co-patching, flourescence correlation spectroscopy (FCS) and variants of FRET, indicate clathrin- and cholesterol-dependent APP-BACE1 co-localization in rafts immediately preceding endocytosis and subsequent processing [102,103,104,105,106,107]. Thus, endosomes are likely the primary site of Aβ production. Endocytic processing of APP is required due to the low pH optimum (~pH 6) of BACE1. In endosomes, where presumably rafts also exist, the BACE1 active site is exposed to the lumen, where the low pH facilitates APP cleavage [108,109]. As with most clathrin-mediated events, much of APP is destined for degradation in lysosomes, with a portion recycling back to the plasma membrane. Insufficient degradation causes Aβ accumulation, a problem hastened by increased basal production of Aβ. Support for such findings came from studies of patients with Down Syndrome (DS), where an extra copy of the APP gene on chromosome 21 invariably results in early onset AD [110]. Ying Jiang and colleagues further attributed the onset of AD in DS patients to endosomal dysfunction that was independent of Aβ levels, possibly reflecting the earliest change in AD pathobiology [111]. Nevertheless, inhibition of BACE1 seemed to correct the endosomal dysfunction in their experiments, thus restoring raft-targeted inhibitors of BACE1 as a plausible intervention. It should be cautioned, however, that the complete knock-out of BACE1 has had untoward side-effects in mouse models, including memory deficits and symptoms that resembled schizophrenia [112]. This may be explained by the many other substrates of BACE1, most importantly neuregulin-1, with roles in CNS synaptic plasticity, myelination, and neurotransmitter modulation [113]. Investigators suggest that partial inhibition of BACE1 may be required, a strategy perhaps sufficient, to prevent Aβ synthesis [114].

## 4. Lipid-based Nanocarriers: Liposomes and Solid Lipid Nanoparticles

Targeted drug delivery to neural rafts may soon be possible, given the rapid advances in nanotechnology (NT). First proposed by Nobel Laureate Richard Feynman in a classic talk presented to the American Physical Society on 29 December, 1959 (“There’s Plenty of Room at the Bottom” [115]), NT is an amalgam of several different fields, e.g., physics, chemistry, molecular biology, and materials engineering, unified (if somewhat uneasily) by the objective of manipulating atomic and molecular phenomena on the nanometer scale, *i.e.*, ranging from one to several hundred nanometers [116,117]. The field is generally divided into “top-down” and “bottom-up” techniques, although the two are sometimes combined. In the former, best exemplified by the semiconductor industry, the process begins with macroscopic material, into which nanoscale details, such as integrated circuits, are incorporated. In the latter technique, exemplified by the synthesis of artificial bone via biomineralization, custom-made molecules are activated to self-assemble by means of a controlled physical “trigger” such as an applied electric field. Formidable engineering obstacles are encountered in both approaches, but NT’s challenges are not limited to apparatus and experimental design. Interdisciplinary communication is an obstacle as well, and is perhaps most pronounced in nanomedicine [118]. A major subdivision of NT, nanomedicine seeks to manipulate molecular processes in human physiology for the diagnosis, treatment, and possible prevention of disease. Recent applications range from iron oxide nanoparticles for use as MRI contrast agents [119], to nanoparticle formulation of tumor suppression gene FUS1 in the treatment of lung cancer [120], to dendrimer-based microbicide gels in HIV prevention [121]. In this section, we will describe the basic properties of two types of lipid-based nanoparticles (LBNs), liposomes and solid lipid nanoparticles, of possible relevance to drug delivery in epilepsy, PD, and AD. It should be noted that these two types are by no means exhaustive, and that several other NT therapeutics are being intensively investigated. For example, NPs composed of biodegradable polymers are being utilized for the targeted delivery of drugs, proteins, nucleic acids, and vaccines [122]. Similarly, silica NPs, which are biocompatible, nontoxic, and porous with modifiable pore size, are being equipped with bioactive molecules such as enzymes, genetic materials, and drugs used in chemotherapy [123]. Our emphasis on liposomes and solid lipid nanoparticles reflects our view that they are more widely deployed in epilepsy, PD, and AD therapeutics than are other NP platforms. Moreover, as with our emphasis on three neural disorders, we thought it best to extensively discuss a small number of NT approaches rather than a large number in summary form. 

### 4.1. Liposomes: Overview

**Figure 2:**
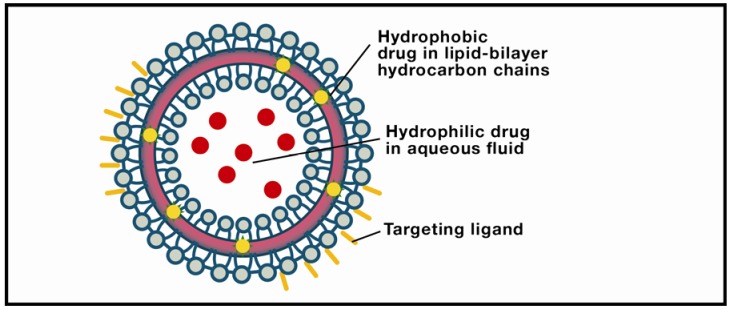
Liposome. The object of intensive research in drug-delivery technology, these structures are typically comprised of a lipid bilayer enclosing an aqueous interior. Solid lipid nanoparticles differ from liposomes by usually having a lipid monolayer shell from which the hydrocarbon chains extend into a solid lipid matrix.

Liposomes show considerable promise for raft-targeting drug delivery due to increasing refinement of fabrication techniques, drug loading efficiencies, ability to be coupled with site-specific targeting ligands, drug release potential, and, most significantly, minimum toxicity. The research platform originated in the early 1950s when hematologist Alec Bangham discovered that phospholipids dispersed in water would self-organize into a bilayer [124]. Subsequent electron microscopy revealed that the dispersion took the form of cell-sized closed vesicles, now called liposomes, suggesting, as Banham noted, a possible vehicle for drug delivery. Today, Bangham’s conjecture has been widely realized due to several attractive features of this pharmaceutical carrier [125,126,127]. Perhaps the most obvious advantage is biocompatibility: The carrier is composed of lipids and water (Figure 2). More exactly, liposomes are spherical, bubble-like structures comprised of one (unilamellar) or multiple (multilamellar) concentric lipid bilayers with an aqueous interior; they range in size from ~20 nm (unilamellar nanocarriers) to ~1–2 µm (multilamellar microcarriers). This structure is pharmacologically versatile: Hydrophilic drugs can be entrapped in the watery interior, while hydrophobic agents can be embedded within the membrane. Moreover, current methods in liposomal synthesis are increasingly overcoming significant physiological challenges. The most intractable of these is the blood-brain-barrier (BBB), a semi-permeable filtering system of capillary endothelial cells, capillary basement membrane, and astrocytic feet which prevents harmful compounds from entering the brain [128]. BBB selectivity is highly optimized: Only lipid-soluble molecules below a threshold of 400–600 Daltons can penetrate the BBB, amounting to less than 2% of small-molecular-weight drugs. However, surface modification of liposomes with vectors that exploit BBB transcytosis, mediated by endothelial-cell receptors and transporters, produces a nanoparticle which can successfully penetrate the barrier. Examples include monoclonal antibodies (mABs), cationized proteins, plasma proteins, and polyethylene glycol (PEG). The latter is widely used because it is biocompatible, has a stabilizing effect on the liposome, and prolongs drug circulation time. PEGylated liposomes have frequently been used in the passive targeting of brain tumors, in which the liposome diffuses through the permeable capillaries associated with inflammation. Active targeting, or site-specific drug delivery, of PEGylated liposomes is achieved by adding a ligand to the liposomal surface or to the PEG terminus. The use of cell-penetrating peptides (CPPs) as ligands shows particular promise due to their ability to translocate across the membrane into the cytosol [129]. Translocation kinetics are not well understood, however; competing theories emphasize direct membrane penetration, and endocytosis-mediated entry. Clearly, a consensus model would expedite the development of more efficient delivery systems. Moreover, new approaches are needed to enhance the timely cytosolic release of the encapsulated drug. One form of active targeting which shows promise for brain therapeutics is the immunoliposome, synthesized by coupling an antibody to the distal tip of the PEG chain. Yun Zhang’s team utilized this technology to deliver a therapeutic gene in a mouse model of intracranial brain cancer [130]. PEGylated liposomes encapsulating a plasmid encoding a short hairpin RNA directed at nucleotides 2529–2557 of epidermal growth factor receptor (EGFR) mRNA suppressed 95% of EGFR function, resulting in an 88% increase in survival time.

Immunoliposome targeting of rafts appears promising, but is in its inception. Rico Gunawan and Debra Auguste actively targeted raft-associated intercellular cell adhesion molecule-1 (ICAM) and endothelial-leukocyte adhesion molecule-1 (ELAM) via liposomes modified with anti-ICAM (aICAM) and anti-ELAM (aELAM) antibodies [131]. ELAM and ICAM are expressed by cardiovascular endothelial cells in response to inflammation, shear, angiogenesis, and atherogenesis (arterial plaque formation). Once expressed, the adhesion molecules mediate leukocyte endothelial contact and subendothelial penetration, critical initial steps in maintaining vascular homeostasis. Earlier studies suggest that ELAM and ICAM clustering on endothelial-cell rafts to facilitate cellular signaling is a feature of the inflammatory response. Based largely on this finding, Gunawan and Auguste proposed that the ELAM-ICAM cluster constitutes a molecular target accessible by immunoliposomes. Using human umbilical vein endothelial cells as an *in vitro* model mimicking cardiovascular endothelium, the investigators targeted ELAM and ICAM receptors using aELAM and aICAM antibodies bound to the immunoliposome surface. Liposomes were prepared from either 1,2-dioleoyl-sn-glycero-3-phosphatidylcholine (DOPC), an unsaturated lipid mobile above the melting temperature T_m_ ≈ −20 °C, or from 1,2-dipalmitoyl-sn-glycero-3-phosphatidylcholine (DPPC) a tightly packed, saturated lipid which forms a gel below T_m_ ≈ 42 °C, to observe the effect of saturation and chain length on antibody mobility. The underlying assumption was that the more mobile aELAM and aICAM antibodies on the DOPC liposomes would settle into a configuration complementing the natural arrangement of the ELAM and ICAM receptors on the targeted cell. The assumption was strongly supported: Binding by DPPC was reduced by more than 2-fold, suggesting that “the mobility of aICAM and aELAM on (DOPC) liposomes may enable clustering, which coordinates with clustering of ICAM and ELAM on endothelial-cell surfaces”. A more generalized implication is the design of “intelligent” liposomes in which mobile surface agents, either antibodies or peptides, would map to raft-associated molecular configurations in a wide variety of cell types including the neuron.

### 4.2. Solid Lipid Nanoparticles: Overview

A more recently developed class of particulate drug carriers is solid lipid nanoparticles (SLNs). This drug-delivery system is defined by its solid lipid matrix (50–1000 nm in diameter) which may contain triglycerides, glyceride mixtures, or waxes which are solid at human body temperature [132,133,134,135,136]. SLNs were first anticipated in the late 1980s when investigators synthesized glyceride-and-phospholipid-based micropellets for oral drug delivery [137]. In these pioneering studies, the micropellets were obtained via spray-drying and spray-congealing in which feed from a fluid state was converted to solid particulate by spraying the feed into a hot drying medium. Current production methods emphasize either hot or cold homogenization. In both methods, the drug is dissolved in the lipid being melted at approximately 5–10 °C above the melting point. The drug-containing lipid melt is then dispersed in a hot surfactant solution by stirring, and the resulting pre-emulsion is passed through a high-pressure homogenizer. The hot lipid nanoemulsion obtained through this process is then cooled, following which the lipid re-crystallizes, forming nanoparticles with a solid matrix. A frequently cited advantage of SLNs is the slow release of the encapsulated drug in contrast with the “burst release” which is a problematic feature of liposomes. However, as Anu Puri’s team has recently cautioned, structural transformation during SLN storage may result in burst release for this class of nanoparticle as well [138]. Rapid nanoemulsion cooling produces a disordered structure, essential for drug loading into the areas of crystal imperfections. But during storage the unstable crystal can convert to the more stable state, a configuration which may result in burst release *in vivo*. Despite this shortcoming, which is the object of much current research, SLNs have several advantages. The carrier is biocompatible, readily traverses the BBB, can incorporate hydrophilic and hydrophobic drugs, has a relatively high drug payload, can be coated with polyethylene glycol (PEG) or PEG-containing surfactants which reduce phagocytosis, and can be coupled with surface ligands, permitting carrier targeting. The last feature is of special significance in the treatment of neural disease.

Although site-specific targeting via SLNs is being vigorously pursued in cancer research (e.g., cell-specific delivery of doxorubicin utilizing a mannose-conjugated SLN [139]), less progress is evident in addressing neural disorders or formulating raft-mediated drug-delivery systems. Instead, most SLN research relevant to brain has focused on surface coatings for overcoming the BBB as a basis for passive targeting [140]. A wide range of surfactants including, in particular, polysorbates 20, 40, 60, and 80, have been proposed as coating materials which could improve brain uptake by modifying surface hydrophobicity. These candidates were compared in a series of experiments evaluating their efficacies in inducing analgesia in mice via dalagin administration [141]. The results indicated that polysorbate 80 (P80) had the highest induction of analgesia, a finding consistent with separate experiments indicating that drugs which do not normally penetrate the BBB (e.g., tubocuarine, loperamide, and doxorubicin) showed higher brain concentrations when P80 coatings were utilized [142,143,144]. Importantly, P80 was used successfully to target tacrine, an anti-AD drug, in an animal model [145]. By implication, site-specific drug delivery in the treatment of AD and other neurodegenerative diseases may ultimately be possible by equipping P80-coated SLNs with targeting ligands.

## 5. LBNs and Raft Targeting in Epilepsy, PD, and AD

### 5.1. Epilepsy

Liposomes and SLNs may be plausible candidates for raft-mediated therapies in epilepsy, PD, and AD. In the case of epilepsy, liposomal approaches initially emphasized delivery of neurotransmitters: Liposomal-entrapped GABA (γ-aminobutyric acid), an inhibitory neurotransmitter once widely researched as a natural defense against epileptic activity, did indeed suppress penicillin- or isoniazid-induced seizures in animal models [146,147]. However, other studies based on the “GABA-deficiency theory of epilepsy” were unsuccessful [148]. For example, treatment with GABA agonists intensified seizures in rats and humans; a similar outcome followed treatment with GABA uptake blockers. As a consequence, current research, so far largely limited to traditional forms of drug delivery not involving nanoparticles, is more strongly focused on anti-epileptic prodrugs, a platform with possible future significance for LBN therapeutics. Prodrugs are medically inactive substances comprised of a pharmacological agent attached to a compound; the latter is removed *in vivo* by hydrolysis or enzymatic cleavage, thereby generating the active form. The rationale is optimization of absorption, distribution, metabolism and excretion of the administered drug. Favorable outcomes in epilepsy treatment have recently been achieved with prodrugs of valproic acid (VPA), phenytoin, and gabapentin [149]. Thus VPA, linked with lecithin, was rendered more lipophilic, facilitating movement across the BBB. Subsequent enzymatic cleavage by phospholipase A_2_’s, highly concentrated at the seizure focus, terminated epileptic activity. Cessation of seizures, in turn, decreased the enzymatic level, thus preventing unnecessary prodrug activation. Feedback mechanisms of this type have significantly increased bioavailability, and have displayed highly encouraging anticonvulsant effects. 

From a more futuristic perspective, LBN-encapsulated AEDs (and, possibly, prodrugs) equipped with cell-penetrating peptides and surface ligands, could potentially be delivered to the seizure focus in a cell-specific manner via targeting of a raft-localized ion-channel isoform (*i.e.*, epilepsy-specific channel subunit) linked to the disease. Research essential for this strategy is still in its formative stages; e.g., it is not yet known if there is a raft-associated NMDAR-channel isoform subunit distinctive to epilepsy. Indeed, regarding this type of approach, Kevin Ogden and Stephen Treynelis, although generally optimistic, caution that “it has been impossible to assess the contribution of specific NMDA receptor subtypes to brain function or evaluate the potential therapeutic utility of targeting particular NMDA receptor subtypes” [150]. The genetic understanding of channel variation, however, has shown remarkable progress. Recently, exomes (coding regions) of 237 ion-channel genes of 152 patients with sporadic idiopathic epilepsy and 139 unaffected, ethnically-matched controls, were sequenced by Jeffrey Noebels’ team as a means of identifying individual mutation profiles signaling elevated risk [151]. The result is a cautionary tale revealing the subtleties of the disease: Deleterious ion-channel mutations do not inevitably produce epilepsy; rather, the effect of any given mutation is dependent on other gene variants with which it is combined. This network viewpoint also applies to the channels: Epilepsy, Noebels proposes, “may arise from a complex mixture of altered channels, and may be prevented by other channels working in the background”. In the light of this study, and the (probably) combinatorially explosive channel-channel interactions implicit in the conclusion, it seems evident that computational modeling of the ion-channel variants is essential not only for understanding epilepsy, but also for devising appropriate molecular therapies. A critical requirement for the latter would be the identification of accessible subunits which could be actively targeted by an LBN (but see cautionary remarks in the PD discussion below regarding LBN targeting of the postsynaptic protein architecture). Nanoparticle translocation into the cytosol would, for example, permit release of TAT-21-40, an NMDAR-binding form of trans-acting activator of transcription (TAT) protein transduction domain (PTD), *i.e.*, TAT-PTD, which may additionally function as a cell-penetrating peptide. Experiments conducted by Ying Zhang’s group (discussed above) demonstrated that TAT-21-40, modeled on the binding site of the DREAM protein for the C0 domain of the NMDAR NR1 subunit, reduces the excessive activation of NMDAR implicated in excitotoxicity [57]. In view of the modulatory interactions among both normal and altered channels proposed in the Noebels study, this or any targeting strategy should be developed in close relation to *in silico* modeling.

### 5.2. Parkinson’s Disease

Research on LBNs for drug delivery in PD has primarily utilized liposomes. Moreover, it resembles the more traditional therapeutic approaches in its emphasis on symptom treatment. The orientation may be conveniently dated from a 1992 animal model in which dopamine-containing liposomes were implanted into the striatum of rats in which unilateral substantia nigra lesions had produced behavioral deficits (asymmetric rotation) [152]. Treatment with apomorphine, which activates dopamine receptors, resulted in partial behavioral recovery. In the years that followed this study, experiments utilizing improved liposome designs made a compelling case for greater liposomal effectiveness in contrast with oral medications. For example, a 1998 rat model in which the effect of liposome-encapsulated dopamine HCl on chlorpromazine-induced symptoms mimicking PD (e.g., catatonia, or neurogenic motor immobility) was contrasted with the efficacy of levodopa market formulations, found that the liposomal approach was superior [153]. In a follow-up chloropromazine study (rat model), the contrasting *in vivo* effects of dopamine HCl-bearing liposomes decorated with glutamate stearylamine versus uncoupled drug-bearing liposomes indicated that the glutamate-coupled liposomes had overall greater therapeutic effectiveness [154].

In parallel with these studies, a separate research platform, more recent and perhaps less visible, has focused on PD’s presymptomatic etiology as a potential target for liposomal drug delivery. Much of this effort has emphasized the role of glutathione (γ-glutamylcysteinylglycine) or GSH, a tripeptide comprised of glutamic acid, cysteine, and glycine, in protecting cells against oxidative damage [155,156]. GSH is synthesized in both neurons and glial cells, but is most highly concentrated in astrocytes. This major subtype of glial cell, found throughout the brain and spinal cord, functions not merely to physically stabilize neurons (an earlier stereotype) but also to mediate the neuron’s physiological and pathological states. Astrocytes release newly-synthesized GSH, via the transporter multidrug-resistance protein 1 (MRP1), into the extracellular space shared with a proximate neuron. GSH is then converted to cysteinylglycine, in turn utilized for neuronal GSH synthesis. Neural defense provided by GSH includes protection against reactive oxygen species (ROS), reactive nitrogen species (RNS), and hydroperoxides. Removal of these toxins generates glutathione disulfide (GSSG) which is reduced back to GSH via glutathione reductase and nicotinamide adenine dinucleotide phosphate (NADPH). In healthy neurons, GSH and GSSG remain in dynamic equilibrium which may be expressed as GSSG:2GSH. However, GSH depletion, for which the cause is presently unknown, leads to increased generation of ROS and RNS, producing oxidative stress which damages the majority of cellular macromolecules, further decreasing the GSH level, and thus completing a destructive loop. A feature of GSH depletion of particular significance for PD is disruption of the ubiquitin-proteasome pathway (UPP), essential for the degradation of misfolded and damaged proteins [157]. This pathway, critical for protein turnover, is an ATP-dependent process in which five ubiquitin monomers are linked to a protein substrate; the complex is then recognized by the 26S proteasome, leading to targeting and degradation of a defective protein. UPP disruption in PD due to GSH depletion leads to accumulation of malfunctioning proteins including, importantly, α-syn (discussed above), ultimately causing death of dopaminergic SN neurons. An intuitively appealing strategy is exogenous administration of GSH; however, only small amounts can cross the BBB, too little to achieve significant therapeutic effect [158]. An indirect strategy involving application of cysteine, one of the three amino acids comprising GSH, is also problematic because cysteine potentially interacts with glutamate receptors, generating excitotoxicity [159]. Based on these limited results, it appears likely that for GSH to be effective in PD treatment, an alternative approach will be necessary.

Could greater success be achieved if glutathione were delivered by liposomes? Gail Zeevalk’s team investigated this question in an elegant *in vitro* study utilizing rat mesencephalic mixed neuronal/glial cultures [160]. GSH intracellular levels were transiently reduced by treatment with diethylmaleate, which chelates with GSH and removes it from the cytosol. Cysteine, glycine, and glutamine (used instead of glutamate to avoid excitotoxicity) were administered to replenish the depleted GSH, and the results were then compared with GSH delivery via liposomes. The difference was dramatic: Liposomal GSH was “100-fold more potent in serving as a source for intracellular GSH repletion”. The group then explored the possibility that GSH-liposomal translocation was due to the neuron’s endosomal-lysosomal system. Treatment with GSH-liposomes was immediately followed by application of endosomal inhibitors phenylarcine oxide (PAO) and concanavalin A (ConA). The use of both inhibitors significantly reduced the utilization of GSH-liposomes. The ConA finding, however, is arguably the more informative; ConA is much less toxic than PAO, suggesting that GSH-liposome reduction accompanying ConA treatment was primarily the result of endosome-lysosome inhibition, rather than compromised cells. Finally the team evaluated possible GSH protection against toxicity, utilizing the herbicide paraquat and the fungicide maneb (PQMB model), both of which induce oxidative stress and are implicated in PD. Mesencephalic preparations were exposed to PQMB “in the presence or absence of various concentrations of liposomal-GSH”. Importantly, the procedure was repeated for dopaminergic neurons within the mesencephalon. In both cases, liposomal-GSH provided dose-dependent protection against oxidative insult, suggesting possible benefit in the treatment of PD. 

Will future therapies also include LBN-based raft targeting? Fabrizio Gardoni’s team has recently suggested that PD-specific subunit variations in NMDARs and the post-synaptic density (PSD), the latter a specialized cytoskeletal region comprised of scaffolding molecules, CAMs, and signaling proteins, present the possibility of pharmacological targeting [161]. Moreover, Tatsuo Suzuki’s group, utilizing liquid chromatography and tandem mass spectrometry, recently detected extensive overlap between protein components of rafts and PSDs [162]. Future research should determine if the structurally variant forms of NMDARs and PSDs believed to be specific to PD are associated with rafts. If this is indeed the case, LBN-based raft targeting utilizing cell-penetrating peptides would be a possible therapeutic approach; it is important to note, however, that much remains to be learned regarding the exact mechanisms of postsynaptic endocytosis. For a brief overview of the architecture by which transmitter receptors and the PSD are linked via the Homer protein to an “endocytic zone” see [163]. The strategy, in outline, would resemble the proposed delivery of TAT-21-40 in the treatment of epilepsy: A PEGylated liposome functionalized with a cell-penetrating peptide and targeting ligand would bear a peptide engineered to inhibit α-syn aggregation, a major factor in PD etiology which appears to be raft-associated. Consistent with this strategy, α-syn inhibitors (ASIs) have been designed by Omar El-Agnaf’s group and found to inhibit fibrillation [164]. The team synthesized a library of overlapping α-syn peptides, each of which was seven amino acids long. By using members of this library to test for binding with full-length α-syn (plus utilizing related studies of the α-syn region critical for fibrillation) the group identified the amino-acid residue sequence α-syn (68–72) as the basis for ASI design. Electron microscopy and thioflavin T (ThT) dye fluorescence, the latter a technique frequently used to quantify fibrillation in the presence of an anti-amyloidogenic compound, indicated that some of the ASI peptides inhibited formation of aggregates; importantly, one peptide, ASI1 (RGGAVVTGR-*NH_2_*) achieved complete inhibition. Thus targeted liposomal delivery of ASI peptides to raft-associated α-syn would appear to be a plausible therapeutic strategy. 

### 5.3. Alzheimer’s Disease

Recently, BACE1 has been a prime target for synthesizing inhibitor drugs [165]. However, due to poor central pharmacodynamic performance, attributable mainly to P-glycoprotein efflux pumps and rapid clearance from the body, no BACE1 inhibitor has reached late-stage clinical trials [166]. A possible solution is the use of advanced liposomes and SLNs for targeted delivery of BACE1 inhibitors to the brain. Simple liposomes containing cholinesterase inhibitors have already been administered to patients with AD. Adding DPPC and cholesterol to the liposome formulation significantly increased the half-life of the drug in the brain [167]. Many obstacles (e.g., site-specific targeting) remain, but the studies show the possibility of placing a small-molecule pro-drug BACE1 inhibitor within a liposome. A number of other studies utilized liposome and SLN coatings with high affinity for Aβ peptide, directing these liposomes toward the site of neurodegeneration. Common within the literature is the use of naturally occurring anti-oxidants to selectively reduce Aβ toxicity. In mice models, the use of quercetin (a flavonoid with anti-oxidant effects) in liposomes effectively bypassed the BBB and limited the extent of cortical and hippocampal degeneration [168]. Consistent with combating the inflammatory aspect of AD neurodegeneration, Mourtas *et al.* synthesized curcumin-decorated liposomes that displayed high affinity for Aβ fibrils. The experimenters found that curcumin in its enol and planar form on the surface of liposomes has anti-fibrillogenic properties [169]. Picone and colleagues synthesized SLNs with the ability to traverse the BBB, and small enough (85–96 nm) to avoid phagocytosis by macrophages. The experimenters entrapped ferrulic acid—another plant-derived anti-oxidant—inside the SLNs and replicated their use in an AD model, resulting in a drop in cellular damage caused by Aβ [170].

A proposed theory of removing Aβ from the brain is sequestration of Aβ in the blood, leading to a “sink effect” that draws Aβ out of the brain via the BBB to counteract the imbalance (similar to Le Chatelier’s principle) [171]. Recently, anti-Aβ monoclonal antibodies were developed for the use in immunoliposomes, demonstrating high affinity to Aβ in the blood [172]. In a separate study, a conformation-specific vaccine was developed against Aβ fragments with a β-sheet structure [173]. Indeed, it has been shown that β-amyloid arranges into β-pleated sheets, a quarternary structure common to all amyloid pathologies, to form the insoluble plaques of AD [174]. Further studies utilizing immunoliposomes are warranted, and may eventually demonstrate efficacy against the amyloid pathology induced by aluminum, while lending credence to the hypothesis that AD is a “conformation-specific” disease [175].

While the above studies represent important steps in bypassing the BBB and possible novel diagnostic tools, none achieved the therapeutic goal of reducing Aβ synthesis in the brain. Following the idea that polyunsaturated fatty acids (PUFAs) play essential roles in neural membrane health, and possibly provide neuroprotection against AD, Eckert and colleagues synthesized unilamellar liposomes containing the omega-3 fatty acid docosahexaenoic acid (DHA) to use in HEK293 cell lines overexpressing APP. The data suggests liposomal DHA increases α-secretase mediated processing of APP, presumably by enhancing membrane fluidity [176]. Separate studies showed that membrane fluidity favors the non-amyloidogenic processing of APP [177,178]. 

In a pioneering experiment by Rajendran *et al.*, a β-secretase inhibitor was linked to a sterol moiety anchored in the plasma membrane. Not only did the team achieve effective inhibition of Aβ production over the free inhibitor, they localized APP and β-secretase to endosomes using green fluorescent protein (GFP). The authors maintain that “by linking the inhibitor to a sterol, we may have not only targeted it to endosomes, but also enriched the inhibitor in raft domains in these compartments”. Rajendran’s team confirmed this hypothesis by using a number of lipid anchors with varying affinities for raft domains, along with FCS and avalanche photodiode (APD) imaging. The authors concluded that “raftophilic anchors of β-secretase inhibitors enhance their inhibitory potential”. These findings were replicated *in vitro* and *in vivo*, suggesting a promising, although challenging, direction in raft-targeted AD drug therapy utilizing LBNs [179].

## 6. Conclusions

While guarding a healthy skepticism, an increasing number of cell biologists regard the *in vivo* existence of rafts as highly plausible. The viewpoint is grounded in converging evidence from multiple techniques including, importantly, single-particle tracking (SPT). Moreover, increased awareness that much of the controversy resulted from a widespread tendency to empirically base raft models on uncorrelated “snapshot” studies, each with a limited time and space scale, is motivating interest in the development of synthetic approaches. Central to the emerging paradigm is the picture of an adaptive 2D ensemble capable of rapid shifts from nanodomains to rafts (10–200 nm) in response to fluctuating extracellular and cytosolic conditions. As this viewpoint gains acceptance, putative raft functional properties are continuing to unfold: Rafts may co-localize proteins and thus facilitate their interaction; they may play significant roles in endocytosis and protein sorting; and they may even, through variations in lipid composition, actively modulate changes in protein shape and function. Can these features be manipulated in drug-delivery systems? In the case of neural disease, the answer is cautiously affirmative, acknowledging the existence of significant technical obstacles. The molecular etiologies of three major neural diseases, epilepsy, Parkinson’s disease (PD), and Alzheimer’s disease (AD), appear to involve raft functional properties or raft localization of pathological proteins, suggesting that these platforms could be actively targeted. Importantly, all three diseases are multi-factorial in nature, and “complete cure” would be virtually impossible through the sole use of raft-targeting strategies. Nonetheless, the possibility of raft targeting therapeutics represents a promising direction in altering pathophysiology, especially since reversal of neurodegeneration is a new possibility that has been insufficiently explored. Lipid-based nanoparticles (LBNs), in particular liposomes and solid lipid nanoparticles, may prove effective in this regard due to the increasing efficiency of their fabrication techniques, their ability to be coupled with cell-penetrating peptides to traverse the blood-brain barrier (BBB), their site-specific accuracy when functionalized with targeting ligands, their drug-release potential, and their limited toxicity. However, formidable obstacles exist: In AD therapeutics, for example, an LBN bearing a sterol-linked β-secretase inhibitor would have to penetrate the BBB, actively target a neuron in which the disease was present, and then fuse with the target membrane to facilitate drug movement into the endosome. Manufacturing of such a vehicle thus confronts highly challenging, perhaps physically incompatible, properties. Future results will indicate if LBN technology can fabricate a carrier in which these functions unfold in a precisely timed sequence. In the short run, the growing appeal of interdisciplinary approaches combined with the encouraging results of computational models continue to yield dividends that may soon make the intractable possible. 
